# Supplementary figures and images for: Drifting codes within a stable coding scheme for working memory
Source: PLoS Biol. 2020 Mar 2;18(3):e3000625. doi: 10.1371/journal.pbio.3000625 (PMC7067474; doi:10.1371/journal.pbio.3000625)

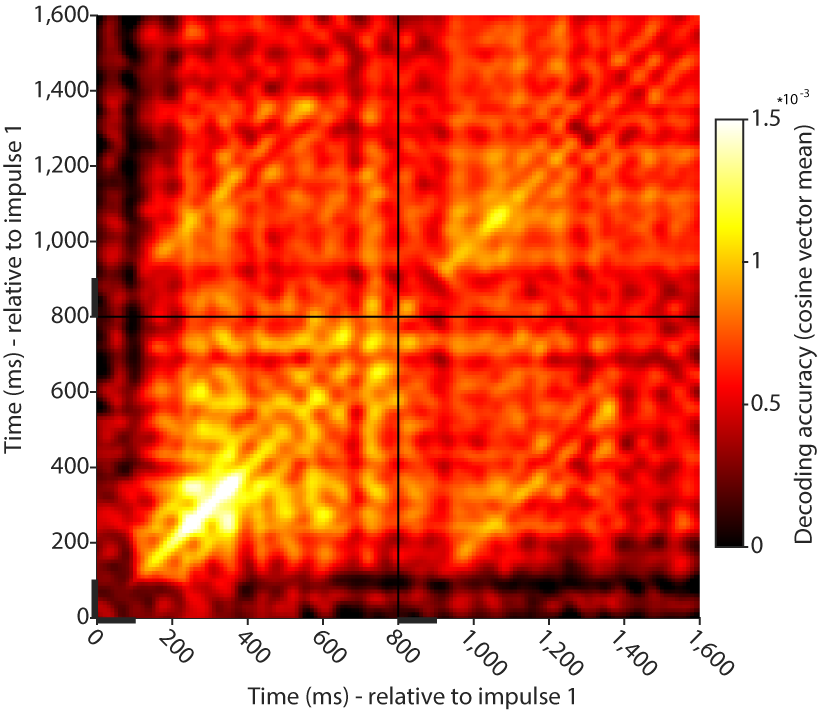

Supplement: S1 Fig — Black bars indicate the presentation times of the impulses. Continuous EEG data from posterior channels (see Methods) were baselined relative to impulse 1 (−200 to 0 ms), smoothed with a gaussian smoothing kernel (SD = 16 ms), and down-sampled to 100 Hz. The classifier (the same as described in the Methods) was then trained and tested on all possible time point–by–time point combinations. Data available at osf.io/cn8zf. EEG, electroencephalography; SD, standard deviation. (TIF) [file pbio.3000625.s001.tif]

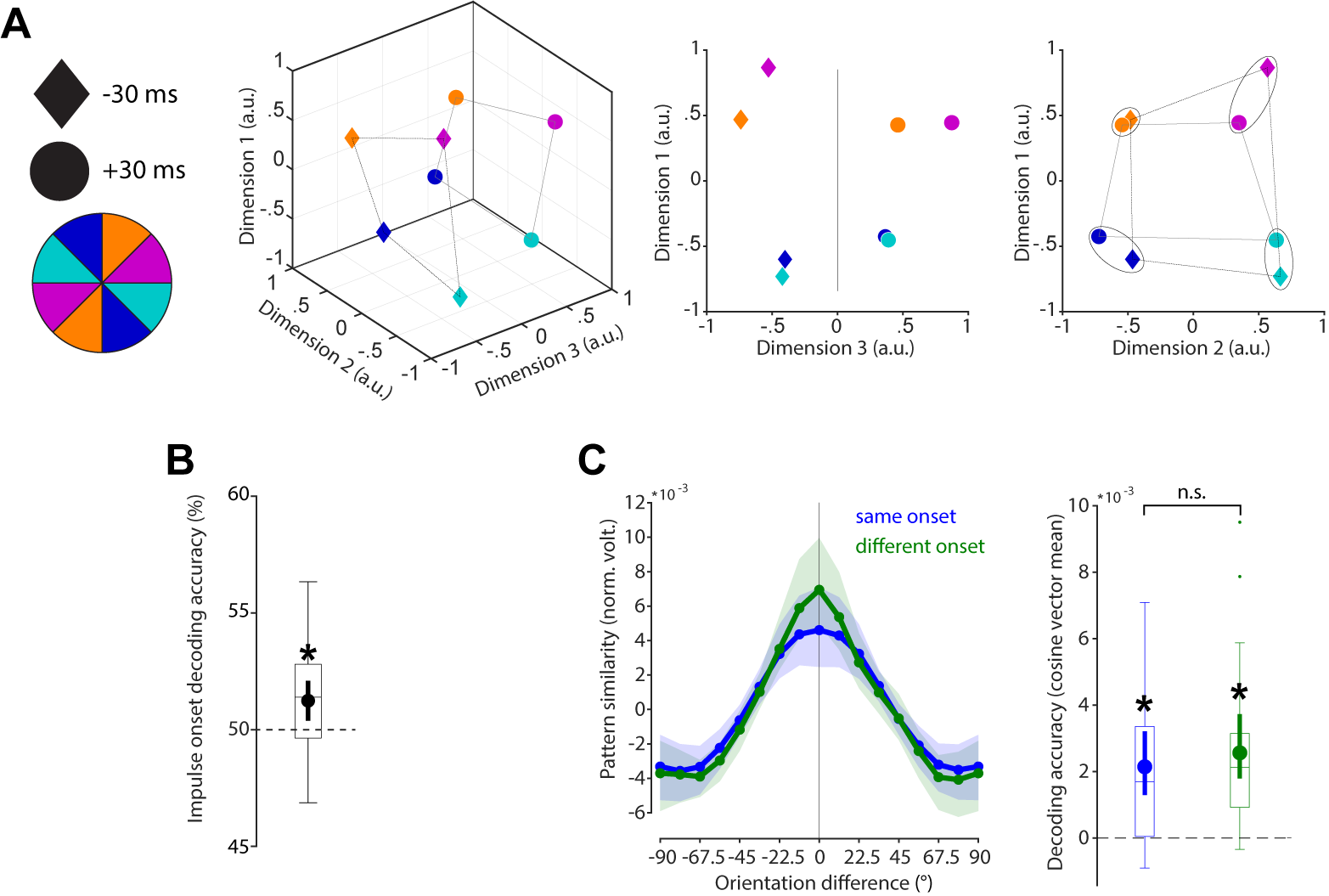

Supplement: S2 Fig — (A) Visualisation of orientation and impulse-onset code in state space. The third dimension discriminates between impulse onsets. The first and second dimensions code the orientation space in both impulses. (B) Trial-wise accuracy (%) of impulse-onset decoding. (C) Orientation decoding within each impulse onset (blue) and orientation code cross-generalising between impulse onsets (green). Error shadings and error bars are 95% CI of the mean. Asterisks indicate significant decoding accuracies or cross-generalisation (p < 0.05). Data available at osf.io/cn8zf. (TIF) [file pbio.3000625.s002.tif]

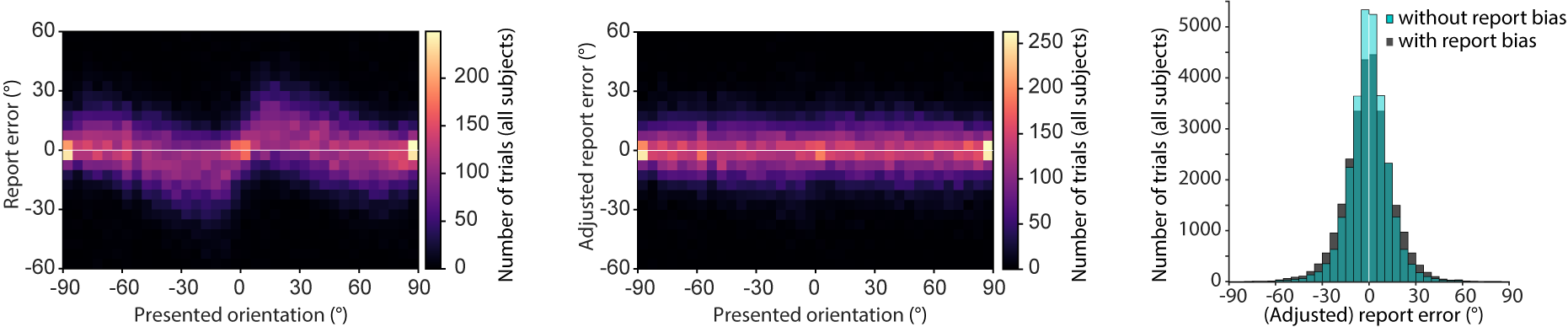

Supplement: S3 Fig — Participants showed a bias, exaggerating the tilt of oblique orientations, manifesting itself as a repulsion form the cardinal axes (0 and 90 degrees; left), similar to previous reports [52]. To ensure an unbiased estimate of a possible shift in our analysis and to isolate random from systematic errors, the report bias was removed by subtracting the median error within 11.25-degree orientation bins (middle). By removing orientation-specific error, the resulting error distribution is narrower (right). Clockwise and counterclockwise reports were defined as positive and negative reports relative to this ‘adjusted’, unbiased report error. Data available at osf.io/cn8zf. (TIF) [file pbio.3000625.s003.tif]

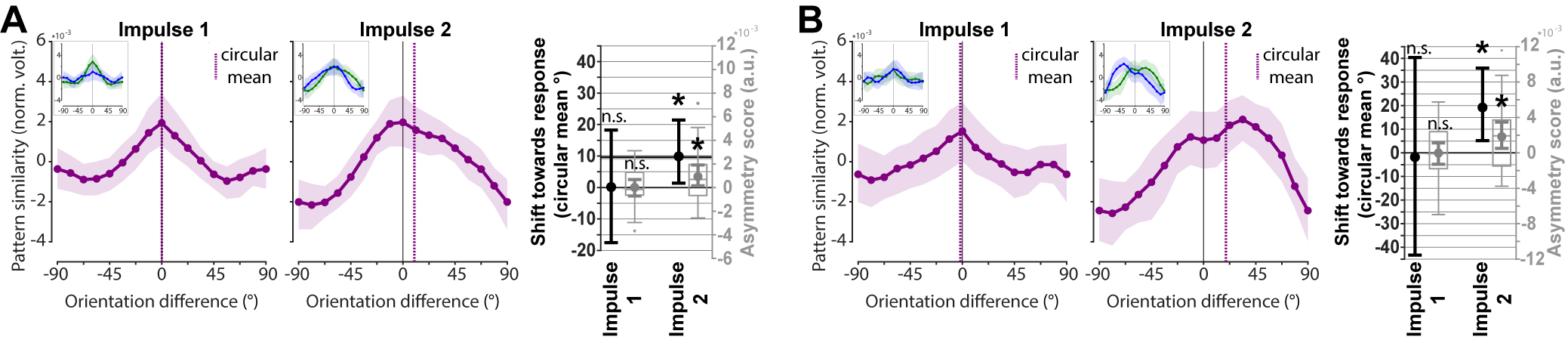

Supplement: S4 Fig — (A) Response-dependent averaging of trial-wise similarity profiles (Fig 6A). Shift towards response: Impulse 1: p = 0.492 (circular mean), p = 0.500 (asymmetry score); Impulse 2: p = 0.022 (circular mean), p = 0.020 (asymmetry score), one-sided. (B) Response-dependent training and testing (Fig 7A). Shift towards response: Impulse 1: p = 0.545 (circular mean), p = 0.525 (asymmetry score); Impulse 2: p = 0.009 (circular mean), p = 0.004 (asymmetry score), one-sided. Same convention as Figs 6B, 6C, 7B and 7C. Data available at osf.io/cn8zf. (TIF) [file pbio.3000625.s004.tif]
