# Supplementary material for: Drifting codes within a stable coding scheme for working memory
Source: PLoS Biol. 2020 Mar 2;18(3):e3000625. doi: 10.1371/journal.pbio.3000625 (PMC7067474; doi:10.1371/journal.pbio.3000625)
Supplement: Attachment — Submitted filename: Response to reviewers_2nd subm_updated.docx [file pbio.3000625.s005.docx]

We would like to thank all reviewers for their thoughtful comments and suggestions.

**Reviewer #1:**

1. Possible typo on p. 9? The text states that the authors focused on windows spanning 100-400 ms after each impulse, then sort the data into 10 ms bins. That should produce 30 bins per impulse and a total of 17 x 30 = 510 dimensions, not the 50 and 850 described in the paper.

***Author response:*** *Yes, indeed this is a typo. We apologise for the confusion and fixed it (line 183).*

2. In collapsing across time the authors' stated goal is to improve statistical power. So, why bother with the 10 ms bins? The authors don't provide a justification for selecting that bin size (nor can I think of one), so I naturally wonder whether key findings generalize if the data are sorted in a different (arbitrary) way. For example, do the authors get the same thing if they average data across the whole 300 ms post-impulse epoch (which would presumably maximize statistical power)? What happens with larger bin sizes (50 ms, 100 ms, etc)?

***Author response:*** *Again, our apologies for not being clearer about this in the manuscript. Although it is possible to simply average the whole time window of interest as the reviewer suggests, this would ignore potentially important neural dynamics within the 300ms epoch. Instead, our approach considers the spatiotemporal dynamics for the full impulse response (see lines 176 to 184). By subtracting out the mean activity over time, this approach provides a measure of the dynamic response that is independent of the absolute voltage baseline (minimising the effect of voltage drift in EEG). We downsample the time-domain to reduce the overall dimensionality, using a 10ms bin size to take advantage of the dynamics below 100Hz. We settled on a relatively conservative bin size as a reasonable comprise between averaging over time points to reduce dimensionality whilst preserving meaningful dynamic neural signals (>100Hz).*

*For all our main analyses, we have adopted a standard approach using 100-400ms, down sampled to 100 hz. These parameters were developed in independent data (reanalysis of a previous study, Wolff et al., 2017), and have since become our default method for decoding orientation with EEG (see also our recent paper in JoN, Wolff et al., 2019). We believe that using a consistent set of parameters across studies helps to avoid potential overfitting.*

3. Eye movements can contribute to EEG decoding (Mostert et al eNeuro 2018; Quax et al NIMG 2019), and small but robust eye position biases have been found in retrocue tasks like the one used here (van Ede et al NHB 2019). The authors went to great pains to minimize effects of eye movements by manually rejecting trials with visible EOG displacements and applying ICA to remove other signals from the residual data set. Still, small-but-consistent eye movements could contribute to decoding performance, for example if a subject moved his or her eyes along the axis of the retrocued orientation. Successful cross-hemifield decoding argues against biases in eye position if one assumes that those biases reflect a mixture of stimulus location and orientation, but a more formal and perhaps compelling approach would be to regress residual horizontal EOG activity onto the orientation of the cued stimulus whether there's any systematic relationship between eye position and voltage -
hopefully not!

***Author response:*** *Indeed, we appreciate the issue raised here. Recent studies have shown that small but systematic deviations in eye position can track the orientation of a centrally presented grating (e.g., Mostert et al eNeuro 2018). As recommended in Mostert et al, we present our stimuli at peripheral locations, minimising the tendency to track orientation. And as the reviewer notes, we also pay close attention to the EOG recordings, rejecting any trial with an observable deviation. However, to address the reviewer’s concern specifically on this point, we have now regressed the residual horizontal EOG activity onto the orientation of the cued stimulus as suggested (see figure below, top row). This showed no systematic relationship between the two. For completeness, we also added both horizontal and vertical EOG into a decoder, and still found no significant evidence for systematic eye movements (bottom row).*

*Top row: Circular correlation of absolute voltage of horizontal EOGs at each time-point (baselined from -200 to 0 ms relative to memory array onset) with the orientation of memory items (in memory array presentation epoch) and the orientation of the cued item (in impulse epochs). Error lines represent 95% CI.*

*Bottom row: Same convention, but time-point by time-point decoding using both horizontal and vertical EOGs.*

**Reviewer #2:**

Major issues

Line 47 (and also line 463):  There are some dynamic codes that can be read out using a simple set of linear weights, as is typically done for stable codes, so saying that dynamic codes “necessarily entails a more complex readout strategy” is not really correct. For example, imagine that there is a perfect synfire chain of neurons where each neuron fires only to one stimulus at one particular point in time. Then a fixed set of weights would work perfectly well for decoding since having the irrelevant weight outside of a neuron’s time-stimulus selective window wouldn’t matter since the neuron would never fire at that time, while they would contribute positively to predictions in their stimulus-time window. However, this type of coding scheme would be considering dynamic by most people (e.g., it would have a strong diagonal pattern in a temporal cross decoding analysis).

Many areas do show this type of sparse sequence coding (see Hahnloser, Kozhevnikov and Fee, Nature 2002), and even for less sparsely firing areas, dynamic neurons often fire at much higher rates during window when they are selective so that their lower firing rates outside these windows would not contribute much noise to any simple linear decoder. There have been some decoding analyses showing that using a fixed set of weights on a dynamic code still can extract most of the information present (see Sreenivasan and D’Esposito Nat Rev Neuro 2019 and Meyers J Neurophys 2018). Changing this sentence and citing these other reviews would be useful.

***Author response:*** *We agree this is an interesting and relevant point. It is often possible to define a single discriminative function that is able to decode a time-varying code at multiple time points. However, the property of dynamic coding we focus on here is temporal generalisation. More specifically, a time-general decoder needs to be learnt by taking all to-be-decoded time points into account to fit the appropriate discriminative function (as discussed in Meyers J Neurophys 2018, and implied in reference to a stable subspace in Sreenivasan and D’Esposito Nat Rev Neuro 2019). However, a decoder trained to decode just at one time point would not generalise to other time points. We believe this has important implications for the representational abstraction of candidate coding schemes (Bernadi et al., bioRxiv 2019)In our study, we find positive evidence of generalisation between different time points during the delay, verifying that the stable subspace can be used to generalise over any time point. In the revised discussion, we shift the focus away from how dynamic coding might ‘complicate readout’ to how the ‘dimensionality of dynamic coding places an important constraint of the generalisability of a particular coding scheme over time’, with more direct reference to the literature noted by the reviewer (lines 507-516).*

Line 174: When the mean activity was subtracted, was this done separately on the training set and then applied to the test set? If not, then data leakage is possible. If it was done separately, it would be good to state this.  Similarly, on line 180, the averaging was done separately for the training and test sets right? Stating this and that none of the same data is in the training and test sets would be good (overall I found the description around line 180 to be confusing).

***Author response:*** *We apologize for the unclear explanations of our methods. We have now extended and clarified the relevant section. The subtraction of the mean activity was done across time and each trial within each channel separately, so there is no data leakage across trials. Furthermore, averaging of trials for each angle bin was only done for the train trials (now stated), not for the independent test trials (lines 176, lines 195-198).*

Finally, it would be good to state that separate trials are used around line 211.

***Author response:*** *We are now explicitly mentioning this (lines 219-222).*

Figure 4. It would be interesting to see the full temporal cross decoding matrix for this and perhaps some of the other results as well, either as a main figure or a supplemental figure. This would be informative to see how long the information is maintained for and how stable the code is, even if there is only information at the time of the pulses.

***Author response:*** *As detailed in our first response to R1, our analysis of the impulse response is based on the spatiotemporal profile within the epoch of interest. The time-stability refers to the generalisation of memory-specific trajectories triggered by the early and late impulses. This improves signal to noise ratio by pooling information over time, whilst allowing for multivariate fluctuations within the time window of interest (evoked dynamics). Our analysis is not optimised for directly comparing spatial patterns at a finer temporal resolution (i.e., within the epoch). Therefore, to address the reviewer’s suggestion, we applied a similar approach to our previous studies to illustrate cross-temporal generalisation within (and between) impulse epochs. For completeness, we now include this in the supplemental (lines 403-405 and supplemental figure 3).*

Also, why is “decoding accuracy used for impulse decoding and “pattern similarity” used for orientation analysis. It would be nice to see both these measures for all types of information as a main or supplemental figure.

***Author response:*** *“Decoding accuracy” is a summary value that denotes how well the classifier performed. When decoding early vs late impulse, this is simply the percentage of correctly labelled impulses (since it is a categorical classification, and which is the same for location decoding in Fig. 5). In the case of orientation decoding, decoding accuracy reflects the “peakiness” of the pattern similarities as a function of orientation difference (i.e. cosine vector mean), since this classification is continuous. We have used this approach in previous work (Wolff et al., 2017) and it is also used for continuous decoding of locations (Sprague et al., 2016). All statistical inferences on decoding performance are based on these decoding measures. We nevertheless also show the pattern similarities for orientations for further illustration. This helps unpack the decoding measure (showing similarity over the orientation difference space), which is not necessary for the binary classifications (as they are more directly based on the pattern difference). To quantify the shift in representation for cw/ccw responses, we also use the pattern-similarity over orientation difference (see Figures 6 & 7). This does not have an obvious decoding counterpart.*

Line 356 (and elsewhere). When you say the errorbars are 95% CIs, are these CIs calculated over the different participants? I don’t think how these CIs are created are described in the methods section.

***Author response:*** *Thank you for pointing this out. We have added the explanation to the methods section (lines 344-345).*

Minor issues

Figure 1. For part A, it would be good to label the axes, and to put the labels for the bar chart below the corresponding bars (or use a color key) to make it clearer what the bars correspond to. Mentioning that for the middle plot that some features are stable and others are dynamic would be useful (and perhaps labeling which is which). For part B it would be good to label the axes and include a color bar.

***Author response:*** *Thank you for these suggestions. We have updated the figure and figure caption accordingly (lines 61-69).*

Line 153: Technically applying ICA across all the data first before doing the cross-validation analyses could lead to data leakage, so if you are manually rejecting eye artifacts anyway, leaving this step out could be better, although in all likelihood this is fine (particularly because if it was data leakage then it would be possible to decoding the unattended stimulus as well).

***Author response:*** *We agree that data leakage is a potential concern if it was condition-specific (i.e., signal sharing between condition-related samples). However, in our experiment, orientation is randomised between trials, and our cross-validation is performed across trials (even when we cross generalise between early or late impulse). Therefore, we are confident that leakage over time (due to ICA, temporal filtering or even noise temporal autocorrelation) would not confound our analysis. Indeed, as the reviewer suggests, there was no significant decoding for the unattended stimulus at the second impulse.*

Line 215: You mean to say when the left stimulus was attended, right? My understanding of the design is that the stimuli were always presented bilaterally. It might also be good to say that fixation is inherently controlled by the fact that eye movements would cause artifacts, since I am assuming there is no other fixation control.

***Author response:*** *Yes, that is indeed what we meant. We have clarified the phrasing of this accordingly and added an explicit mention about the exclusion of eye-movements in the procedure and pre-processing sections (lines 118, 155, 223-226).*

Line 279: Would this analysis be more sensitive if the magnitude of the mistakes was taken into account?

***Author response:*** *This is an interesting point. We have not tried this, but we believe that it will be difficult to balance trial-numbers as a function of magnitude. The necessary sub-sampling and further splitting of trials across several error magnitudes will result in fewer trials in each training set, which will likely reduce power.*

Line 310: Before describing the results, it would be useful to reiterate the some of the rationale of the study.

***Author response:*** *We have now added a short description (lines 350-351).*

Line 315: with a p-value around 0.057 it could just be a lack of power for the reason you don’t see information about the uncued item. The fact that the curve looks tuned to the correct orientation and that there is no statistically significant difference between the cued and uncued location also suggests this is the case.

***Author response:*** *This is a good point. We have updated the interpretation of the results accordingly (lines 364-368).*

Figure 3 is very nice. I don’t think you need to explain what a boxplot is in the caption in this or any of the other figures since it is a pretty common type of plot.

***Author response:*** *We have removed the description of the boxplots from the figures.*

Paragraph 365-375. I don’t think one would expect too many dynamics in a short window of 100 ms difference.  I think this paragraph can be removed.

***Author response****: The main point of running this additional analysis was specifically to test for low-dimensional time-differences in the absence of impulse order effects (in the main experiment, both early and late impulses were presented on each trial). We agree with the reviewer that one would expect minimal differences in the actual coding scheme at such a fine time scale, but this was not the main focus of this result. Rather, we believe that replication of a low-dimensional temporal dynamic in this separate experiment provides important further insights.*

Line 379. It is a very interesting finding that the code in EEG is stable. It would be good to mention that this is at a more global scale of brain networks that EEG picks up and doesn’t necessarily mean there is a stable pattern at the level of neurons.

***Author response:*** *We have now acknowledged this point (lines 420-422).*

Line 384. It is interesting that the orientation information appears to be a lower level visual signal that don’t generalize across locations. Perhaps orientation information would generalize across position in PFC but can’t be picked up by EEG due to the fact that there is less retinotopy in PFC?

***Author response:*** *We have now acknowledged this in lines 432-434.*

Line 466. The amount of dynamics might depend on the task. Remembering a stimulus orientation is a simple task (no transformation of information needed) which might explain why the code is stable.

***Author response:*** *We have now acknowledged this (lines 525-526).*

Line 494: Might be better to say that drifts along the orientation dimension are “correlated” with recall errors rather than “predicted” since you didn’t try to predict errors on individual trials (although it would be neat if you tried to do that).

***Author response:*** *We have updated the phrasing throughout the paper accordingly.*

**Reviewer #3:**

1.    In some analyses (the ‘orientation reconstruction’ analysis), the authors use 16 orientation bins (e.g., Fig. 3), while for the MDS visualizations (e.g., Fig. 4A), they use 4 – are similar conclusions supported with a more granular form of the analysis – maybe with 8 or 16 bins?

***Author response:*** *To clarify, the MDS is simply for visualization. To maximise the number of trials in each bin, we used the minimum number of bins to visualize a circle (i.e. four). The actual statistical analyses are done on 16 bins as usual.*

2.    Similarly, the authors smooth the ‘orientation reconstructions’ with a kernel derived from a cosine raised to a high power. How do results look without the smoothing kernel applied? I know the quantification should not change, as the smoothing kernel will not impact the decoding accuracy or circular bias measures reported, but I’m curious what the data look like when no smoothing is used.

***Author response:*** *As stated by the reviewer, smoothing does not artificially inflate our decoding measure. If there is no parametric structure to the data, then there is no benefit. Therefore, we believe it is a valid processing step for our analysis. For completeness, we present the unsmoothed reconstructions for the reviewer here.*

*Unsmoothed equivalent of figure 3.*

*Unsmoothed equivalent of figure 6.*

*Unsmoothed equivalent of figure 7.*

3.    Previous studies have used induced alpha power to assay spatial working memory representations (e.g., Foster et al, 2016; Ester et al, 2018). I understand the authors are not employing a spatial task here, but I’m curious if they’ve tried using alpha power induced throughout the delay interval (not just in response to the impulses) when analyzing WM representations. It’s certainly possible participants are co-opting a spatial strategy, which would invoke an alpha-related mechanism. (Alternatively, because the stimuli were presented laterally, alpha could carry information about the spatial origin of the WM stimulus throughout the delay; e.g., Foster et al, 2017, Current Biology). In any case, I’m curious how the stability of an alpha-based code, if there is one in this task, compares with the orthogonally-dynamic code in the impulse-evoked responses (and/or, how the authors consider such induced alpha WM codes in context of these results).

***Author response:*** *As requested by the reviewer, we present the cross-temporal decoding matrices using induced alpha (10 Hz) here (see below, blue lines represent cue, impulse 1, and impulse 2 onsets).*

*The results are indeed intriguing, as it seems that the cued item’s orientation can be decoded (while the strong cued location is not surprising). However, the present study was not designed for the analysis of continuous oscillations, as the evoked impulse responses quench alpha power, making it difficult to interpret the results (though they seem relatively stable). We thus believe that this analysis is beyond the scope of the current study but thank the reviewer for the suggestion.*

4.    I applaud the authors for employing non-parametric permutation-based statistics throughout the manuscript. However, the tests they use do not seem to take into account the variability in the sample when evaluating significance. For example, when evaluating whether decoding accuracy was above chance, they randomly flipped the sign of the average decoding accuracy (or, equivalently, the orientation reconstruction) and computed a new average across participants 100,000 times, providing a ‘null’ distribution of average decoding accuracies against which to compare the actual value. However, comparing an average to a distribution of averages isn’t extremely meaningful without also considering the variability across participants. As an example, a typical inferential statistic, like a T-test, computes an estimate of difference between two means, divided by a measure of the variability across the sample. Under normality, etc, assumptions, a T-score can be used to estimate a p-value. The p-values computed in this report seem to consider only a null distribution of mean values, rather than a null distribution of mean values compared to their variability. So, I’d suggest instead generating something like a null T distribution (compute a T-score across participants for each permutation iteration, and compare a final T score to the null T distribution rather than the parametric T distribution).

Additionally, for evaluating whether decoding accuracy is above chance, it’s often useful to estimate a decode w/ a “null” training set – shuffle trial labels at model estimation and use this null decoder to recover orientation reconstructions, and compare resulting metrics of decoding accuracy across participants from a distribution of results between a null and intact decoder. (together with a variability-based metric, like null T distribution of shuffled decoding accuracies vs decoding accuracy computed using intact labels). Similar adjustments to
statistical methods can be made to the other analyses employed.

***Author response:*** *We now perform the suggested analysis (described in lines 312-334) and updated all statistical results. None of the statistical results changed qualitatively. The shift analysis is based on the circular mean at the group level (i.e. take average of all tuning curves, then compute mean), therefore we do not have the option to perform the t-test variant of the test. However, we have now added an “asymmetry score” analysis that is computed for each subject separately, where the difference between the pattern-similarities of positive and negative orientation differences is computed to assess a shift towards the response (explained in more detail in lines 298-310). To test for statistical significance, the labels of report errors (instead of the orientation values) were shuffled. Again, this results in the same pattern of results.*

5.    At the beginning of the Results (lines 311-312), the authors conclude that they recovered “parametric information about the presented orientations”, with similar conclusions reached throughout the Results. However, I’m not sure any of the statistical analyses employed conclusively demonstrate ‘parametric’ coding. The visualized reconstructions have been smoothed by a cosine kernel, and the statistical tests seem to be using the “decoding accuracy” metric, which only identifies significant information overall, not the ‘shape’ of that information. (for example, that metric would be reliably positive if there was one large value at the center orientation, and equally-small values at all other orientations). The authors should clarify the basis for these statements, and/or add a direct test of the ‘smoothness’ of the representations.

***Author response:*** *Indeed, we never explicitly test if the coding is parametric. The decoder simply takes advantage of the parametric structure (if it is there) but doesn’t require it. We have therefore removed all references to parametric information.*

6.    Line 326-327, the authors conclude a difference between contralateral vs ipsilateral sites in Fig. 3 for the Memory Array, but not for the two impulses, but this doesn’t seem to be supported by a statistical test.

***Author response:*** *We have now included an explicit statistical test (lines 371-376).*

Minor:
1.    Fig. 1A – what are the dimensions meant to be in these sketches? Neurons?

***Author response:*** *We edited the caption to indicate that these are example state-space plots, where each axis shows activity along an extracted dimensions (e.g., from PCA, based on a set of weights over neurons or sensors).*

2.    Line 110 – I don’t believe an orientation error can be greater than 90 degrees

***Author response:*** *Good catch, thank you. We have corrected it to 45 degrees.*

3.    A few places (like line 409), the authors describe their decoding results as “orientation tuning curve(s)” – I’m not sure this is the best nomenclature to use, since tuning curves/function most typically describes the feature-dependent response properties of recorded neurons. This is instead a somewhat different result – a function describing the relative multivariate distance between an observed activity pattern and lots of other prototype activity patterns.

***Author response:*** *We have changed the nomenclature to “similarity profile” throughout the paper.*

4.    Fig. 6C, 7C – throughout the other figures, the authors use box plots to elegantly and transparently report their decoding results, but these figures use more typical means/error bars – can box plots be used here too? Or, due to the circular nature of the data, is this not possible?

***Author response:*** *Indeed, box plots are not possible here, since circular means are computed at the group level. However, as mentioned above, we have added the “asymmetry score”, which computes the shift at the subject level. We are now showing the boxplots of this measure in the same figures.*
